# The Neuroprotective potentiality of β‐hydroxy β‐methyl butyrate in an Alzheimer's disease mouse model

**DOI:** 10.1002/alz70859_098487

**Published:** 2025-12-25

**Authors:** Ramesh Kumar Paidi, Kalipada Pahan

**Affiliations:** ^1^ Jesse Brown VA Medical Center, Chicago, IL USA; ^2^ Rush University Medical Center, Chicago, IL USA

## Abstract

**Background:**

There is currently no cure for Alzheimer's disease (AD), the most debilitating and complex neurological condition. Alzheimer's disease has no known exact cause; however, it is recognized that several behavioral, genetic, and environmental factors significantly contribute to its development. The main characteristic features of AD are Senile plaques, neurofibrillary tangles, and neuronal loss. Nevertheless, one of the main neuropathology associated with Alzheimer's disease is synapse loss. β‐hydroxy β‐methyl butyrate (HMB) is a typical supplement used by bodybuilders to increase the strength and muscle growth brought on by training. HMB is a safe supplement that doesn't cause any negative side effects, even after prolonged use.

**Method:**

We began by validating HMB's impact on neuronal plasticity and neuronal markers in primary mouse hippocampal neuronal cells. We also administered oral HMB to six‐month‐old XFAD mice for 30 days. We evaluated their cognitive abilities specifically emphasizing long‐term and short‐term memories using the T‐Maze, Barnes Maze, Novel Object Recognition test, and locomotor tests. Western blotting and immunohistochemistry were used to assess in vivo pathway inductions.

**Result:**

Brain neuronal cells from the hippocampus exhibited elevated levels of the neuronal plasticity markers Glut N2A, Glu‐A1, Snap 25, and PSD 95 after being treated with HMB. Oral HMB administration improves cognitive performance in 5xFAD AD mice. SNAP25 and PSD 95 expressions increased after the HMB administration. This was consistent with CREB phosphorylation and enhanced calcium influx in the hippocampus area, which is linked to improved synaptic plasticity.

**Conclusion:**

According to our findings, HMB promotes synaptic protein expression and regulates CREB phosphorylation, potentially regulating brain plasticity and leading to cognitive recovery in AD models. Finally, our results suggested that the HMB is a strong and safe supplement, more investigation on its potential benefits for treating Alzheimer's appears to be desirable.

